# *N*-3-Hydroxy Dodecanoyl-DL-homoserine Lactone (OH-dDHL) Triggers Apoptosis of Bone Marrow-Derived Macrophages through the ER- and Mitochondria-Mediated Pathways

**DOI:** 10.3390/ijms22147565

**Published:** 2021-07-15

**Authors:** Kyungho Woo, Dong Ho Kim, Man Hwan Oh, Ho Sung Park, Chul Hee Choi

**Affiliations:** 1 Department of Microbiology and Medical Science, Chungnam National University School of Medicine, Daejeon 35015, Korea; khwoo1991@gmail.com (K.W.); kiyou1553@gmail.com (D.H.K.); 89hos701@gmail.com (H.S.P.); 2 Department of Microbiology, Dankook University, Cheonan 31116, Korea; yy1091kwak@gmail.com

**Keywords:** *A. nosocomialis*, quorum sensing, apoptosis, OH-dDHL, virulence

## Abstract

Quorum sensing of *Acinetobacter nosocomialis* for cell-to-cell communication produces *N*-3-hydroxy dodecanoyl-DL-homoserine lactone (OH-dDHL) by an AnoR/I two-component system. However, OH-dDHL-driven apoptotic mechanisms in hosts have not been clearly defined. Here, we investigated the induction of apoptosis signaling pathways in bone marrow-derived macrophages treated with synthetic OH-dDHL. Moreover, the quorum-sensing system for virulence regulation was evaluated in vivo using wild-type and *anoI*-deletion mutant strains. OH-dDHL decreased the viability of macrophage and epithelial cells in dose- and time-dependent manners. OH-dDHL induced Ca^2+^ efflux and caspase-12 activation by ER stress transmembrane protein (IRE1 and ATF6a p50) aggregation and induced mitochondrial dysfunction through reactive oxygen species (ROS) production, which caused cytochrome c to leak. Pretreatment with a pan-caspase inhibitor reduced caspase-3, -8, and -9, which were activated by OH-dDHL. Pro-inflammatory cytokine and paraoxonase-2 (PON2) gene expression were increased by OH-dDHL. We showed that the *anoI*-deletion mutant strains have less intracellular invasion compared to the wild-type strain, and their virulence, such as colonization and dissemination, was decreased in vivo. Consequently, these findings revealed that OH-dDHL, as a virulence factor, contributes to bacterial infection and survival as well as the modification of host responses in the early stages of infection.

## 1. Introduction

Several *Acinetobacter* species, such as *A. baumannii*, *A. nosocomialis*, and *A. pitti*, have emerged as clinically significant in nosocomial infections and antibiotic resistance [1,2]. *A. baumannii* and *A. nosocomialis* are common causative agents of pneumonia in hospitals and constitute a large proportion of *Acinetobacter*-species pneumonia patients [3]. Although an *A. baumannii* infection has a higher mortality rate than *A. nosocomialis*, *A. nosocomialis* has recently been reported as increasingly being involved in clinical outbreaks and nosocomial infections [3,4]. However, the specific virulence factors of this microorganism have not been well-characterized.

Quorum sensing (QS) is a communication mechanism that bacteria use to monitor their cell density in order to regulate biofilm formation, virulence factor expression, and survival under stress conditions in different environments [5]. The QS of Gram-negative bacteria is regulated by the LuxI/LuxR regulatory system, which produces *N*-acyl-homoserine lactone (AHL) [6,7]. The Las and Rhl systems of *P. aeruginosa* and the AbaI/R system of *A. baumannii* belong to the LuxR/I family [5,6]. In *Acinetobacter* species, AbaI synthesizes *N*-acyl-homoserine lactone as autoinducer synthases and AbaR regulates the AHL synthesis of AbaI through an AbaR-AHL complex formation that binds to specific promoter sequences as autoinducer receptors (lux-box) [5,8]. Although *Acinetobacter* species generate various acyl chain lengths of QS signal molecules, *A. baumannii* and *A. nosocomialis* generate prime *N*-(3-hydroxy dodecanoyl)-L-homoserine lactone (OH-dDHL) [8,9,10]. Studies have reported that AHL of the lengths C14 and C16 has been detected in *Acinetobacter* clinical strains; however, production differences may occur depending on the growth conditions in the laboratory [8,11].

Various pathogens induce host cell death through virulence factors to evade immune responses and to survive [12,13]. Virulence factors of *A. baumannii* are well-known and include the outer membrane components, capsule, lipopolysaccharides, phospholipases, metal acquisition systems, and protein secretion systems [14,15]. In particular, the outer membrane protein A and outer membrane vesicles of both *A. baumannii* and *A. nosocomialis* have been reported to induce apoptosis of host cells [16,17,18]. Caspase activation in apoptosis plays a central role in cell death, differentiation, proliferation, and the intrinsic and extrinsic pathways and is involved in morphological features such as DNA fragmentation, cell contraction, and membrane blebbing [19]. In the intrinsic pathway, mitochondrial membrane potential (ΔΨm) and the release of cytochrome c from mitochondria also have important roles in the apoptosis mechanism [19,20]. In addition, reactive oxygen species (ROS) are related to the collapse of the mitochondrial membrane potential (ΔΨm) and the release of cytochrome c, which activates caspase-9 and -3 sequentially [21]. The endoplasmic reticulum (ER) functions as an intracellular calcium storage, promotes cell survival and cell maintenance, and is involved in triggering apoptosis under stress conditions [22]. Calcium ions (Ca^2+^) and caspase-12 triggered by ER stress are involved in apoptosis mechanisms mediated through caspase-9 and the release of cytochrome c from mitochondria [23].

Previous reports revealed that the QS molecule *N*-oxo-dodecanoyl-L-homoserine lactone, which contributes to the pathogenicity of *P. aeruginosa*, induces the intrinsic apoptosis of lymphocyte, macrophage, and epithelial cells, whereas *N*-butanoyl-l-homoserine lactone does not [24,25]. Caspase-mediated apoptosis in response to the OH-dDHL released by *Acinetobacter* species is not clearly established. Although the *abaI* mutant strain of *A. baumannii* reported decreased biofilm formation and motility [26], the in vivo colonization and dissemination of the *A. nosocomialis anoI*-deletion mutant strain remains to be identified. In this study, we investigated the induction of an ER-mediated apoptosis pathway with caspase-12 and Ca^2+^ release and a mitochondrial-mediated pathway by OH-dDHL in macrophages. In addition, the virulence of the *anoI*-deletion mutant strain in vivo was examined. We demonstrated that OH-dDHL triggers apoptosis via the ER- and mitochondria-mediated pathways and through oxidative stress in bone marrow-derived macrophages (BMDM), and that the *anoI*-deletion mutant strain decreased the pathogenicity compared to the *A. nosocomialis* wild-type strain.

## 2. Results

### 2.1. Effect of AHLs on Cell Viability of Host Cells

**Figure 1:**
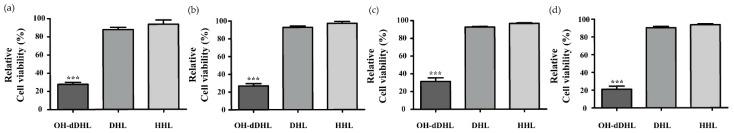
Effect of C6-HSL (HHL), C10-HSL (DHL), and OH-dDHL on the cell viability of host cells. (**a**) BMDMs were treated with 50 µM AHLs for 24 h. (**b**) THP-1 cells, (**c**) A549 cells, and (**d**) HEp-2 cells were treated with 100 µM AHLs for 24 h and cell viability was measured with the CCK assay. Data are presented as mean ± SEM of three independent experiments. ***, *p* < 0.001 vs. DHL and HHL.

**Figure 2:**
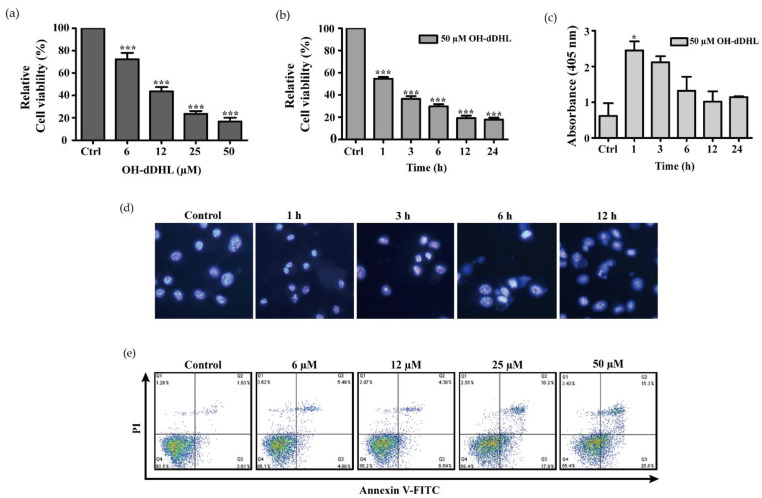
OH-dDHL-induced apoptosis in BMDMs. (**a**,**b**) Cell viability was determined using CCK assay. BMDMs were treated with OH-dDHL in a time- and dose-dependent manner for 24 h. (**c**) DNA fragmentation was analyzed using a Cell Death Detection ELISA kit in 50 µM OH-dDHL-treated BMDMs in a time-dependent manner. Data are presented as mean ± SEM of three independent experiments. ***, *p* < 0.001, *, *p* < 0.05 vs. control. (**d**) Apoptotic cells were stained with DAPI. Images were captured using a fluorescence microscope. Magnification: ×400. (**e**) Apoptotic population was stained with Annexin V/PI and detected using Flow cytometry.

In previous reports, many *Acinetobacter* species showed different AHL profiles, and none of the AHL signals could be specifically assigned to species of the genus *Acinetobacter* [11]. We selected three types of AHL that are representative of the *Acinetobacter* species and investigated the cell viability of the host cells depending on the type of AHL [10,11]. Both *N*-hexanoyl-DL-homoserine lactone (C6-HSL: HHL) and *N*-decanoyl-DL-homoserine lactone (C10-HSL: DHL) slightly impacted cell viability in the primary cell, monocyte, and epithelial cell lines (Figure 1). However, OH-dDHL decreased the viability of the host cells by >70% (Figure 1). In addition, cell viability with OH-dDHL decreased in a time- and dose-dependent manner for BMDM (Figure 2a,b) and the same results were shown for A549 cells, HEp-2 cells, and THP-1 cells (Figure S1). To further determine the potential role of OH-dDHL in regulating the viability of host cells, we treated BMDM cells with OH-dDHL at different time points and then analyzed the typical morphological features through DAPI staining, DNA fragmentation analysis, and Annexin V/PI double staining. Cell death, such as DNA fragmentation, cellular shrinkage, round-up, and detachment from the culture plate and nuclear condensation were clearly identified by morphological analysis after 6 h by 50 µM of OH-dDHL (Figure 2c,d). Furthermore, OH-dDHL showed significantly increased effects on the death of cells that were treated with OH-dDHL in a dose-dependent manner (Figure 2e). These results revealed that OH-dDHL induces the apoptosis of host cells in a dose- and time-dependent manner.

### 2.2. OH-dDHL Induced Cell Death by Caspase Activation

**Figure 3:**
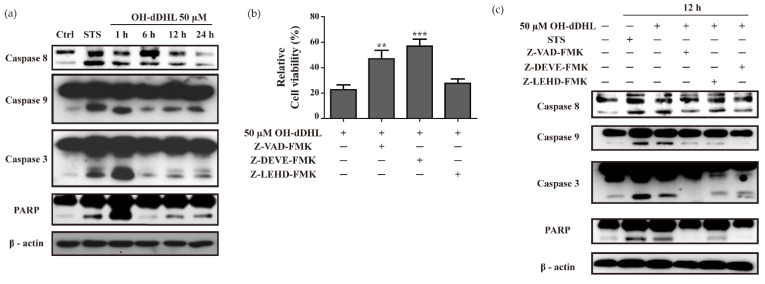
OH-dDHL-induced caspase activation. (**a**) BMDMs were treated in a time-dependent manner. Staurosporine (STS) was used as a positive control. Caspase-8, -9, and -3, and PARP were analyzed using Western blot. Ctrl; negative control. (**b**) Caspase inhibitor 30 µM Z-VAD-FMK, 40 µM Z-DEVE-FMK, 30 µM Z-LEHD-FMK pretreated BMDMs were treated with 50 µM OH-dDHL and cell viability was measured with CCK assay. Data are presented as means ± SEM of three independent experiments. ***, *p*< 0.001, **, *p*< 0.01 vs. OH-dDHL. (**c**) Western blot of caspase-8, -9, and -3, and PARP in caspase inhibitor-pretreated BMDMs with 50 µM OH-dDHL.

AHL’s ability to trigger cell-death-related events when released by *P. aeruginosa* has been previously reported [27], but it is not known whether it is associated with apoptosis signaling by *Acinetobacter* species. Since the induction of apoptosis is the most well-known caspase-dependent activation of programmed cell death [19], we investigated whether OH-dDHL-induced apoptosis occurs due to caspase-activation cascades in BMDM cells. For this purpose, we performed time-course experiments of the caspase cascade for up to 24 h at 50 µM of OH-DHL. The increases in the cleaved forms of caspase-8, -9, and -3 and PARP were most pronounced within 1 h by OH-dDHL on BMDMs and remained elevated for up to 24 h (Figure 3a). We also analyzed the caspase-activity of A549 cells by adding 100 µM OH-dDHL and cleaved caspase-8, -9, and -3 were increased as shown in Figure S2.

To further determine whether OH-dDHL-induced apoptosis was inhibited by caspase inhibitors such as Z-VAD-FMK (pan-caspase inhibitor), Z-DEVE-FMK (caspase-3 inhibitor), and Z-LEHD-FMK (caspase-9 inhibitor), these were applied to the cells for 1 h before OH-dDHL treatment. Pan-caspase inhibitor and caspase-3 inhibitor restrained apoptosis >15% with OH-dDHL, but caspase-9 inhibitor showed no significant inhibitory effect (Figure 3b). All the caspase inhibitors partially or completely inhibited caspase and PARP activation of the OH-dDHL-treated cells (Figure 3c). These results demonstrate that OH-dDHL induced the caspase activating cascade by intrinsic and extrinsic pathways and suggest that OH-dDHL could cause mitochondria-dependent and -independent apoptosis in the BMDMs.

### 2.3. OH-dDHL Induced ER Stress Response in BMDM

**Figure 4:**
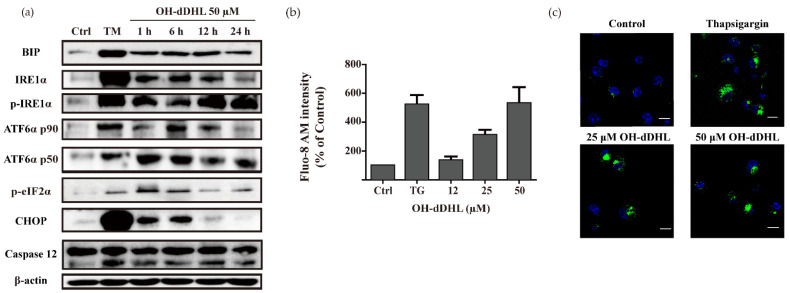
OH-dDHL-induced apoptosis through ER stress. (**a**) BMDMs were treated with 50 µM OH-dDHL in a time-dependent manner. All signal molecules were analyzed using Western blot. Tunicamycin (TM) was used as a positive control. Ctrl; negative control. (**b**) BMDMs were stained with Fluo-8 AM for 1 h and treated with OH-dDHL in a dose-dependent manner. The fluorescence signals were measured at Em = 520 nm. Thapsigargin (TG) was used as a positive control. Data are presented as mean ± SEM of three independent experiments. (**c**) BMDMs with OH-dDHL for 1 h were incubated with Fluo-8 AM in HBSS (Hanks’ Balanced Salt Solution) at 37 °C, 5% CO_2_ incubator for 1 h and were imaged with a confocal microscope using the FITC channel. Scale bar, 10 µm.

The endoplasmic reticulum (ER) is the primary organ responsible for the proper folding and processing of early protein and Ca^2+^ homeostasis [22]. Disrupted ER function leads to a condition known as ER stress [22]. However, sustained and severe ER stresses lead to cell death via apoptosis [19]. To investigate whether OH-dDHL induces an ER stress response, we checked the production levels of the ER stress markers BIP, IRE1α, ATF6α (p90, p50), p-eIF2α, CHOP, and caspase-12 in BMDMs over time via immunoblotting. OH-dDHL treatment strongly induced the production of ER stress-sensor molecules in the UPR and ER, such as BIP, IRE1, ATF6, as well as the phosphorylation of eIF2α; these proteins’ levels were increased after 1 h of OH-dDHL treatment in BMDMs (Figure 4a). In particular, CHOP and caspase-12, which are associated with apoptosis, were remarkably activated. The production levels of CHOP were shown to be affected in BMDMs by OH-dDHL in a time-dependent manner, with a maximal increase observed after a 1-h and 6-h incubation (Figure 4a). In addition, the levels of pro-caspase-12 were not significantly altered but the level of the active form caspase-12 was slightly increased until 12 h of OH-dDHL treatment, returning to early time values at 24 h. The cleaved caspase-9, -3, and PARP were correlated with this result, as observed in OH-dDHL-treated cells after a 1-h incubation (Figure 3a). These results indicated that the activation of CHOP under OH-dDHL induced ER stress conditions and decreased the mitochondrial membrane potential, leading to cytochrome c release. The OH-dDHL-induced ER stress led to cell death in BMDMs mediated by caspase-12 activation (mitochondria-independent pathway) but also through the mitochondria-dependent pathway that relies on cytochrome c release and the subsequent activation of caspase-9 and -3, leading to the cleavage of apoptosis substrates.

Next, we investigated whether the cytoplasmic free calcium concentration is affected by OH-dDHL. The cells were stained using Fluo-8 AM. As shown in Figure 4b,c, the calcium concentration of the cells was significantly higher than that in the control and over-loaded calcium was determined using a confocal microscope, suggesting that OH-dDHL could induce cytoplasmic calcium overload and, thus, induce cell damage. These results suggest that OH-dDHL induces cytoplasmic free calcium through the depletion of the ER calcium store and the activation of the unfolded protein response.

### 2.4. OH-dDHL Induced ROS Production and Mitochondrial Dysfunction in BMDM

**Figure 5:**
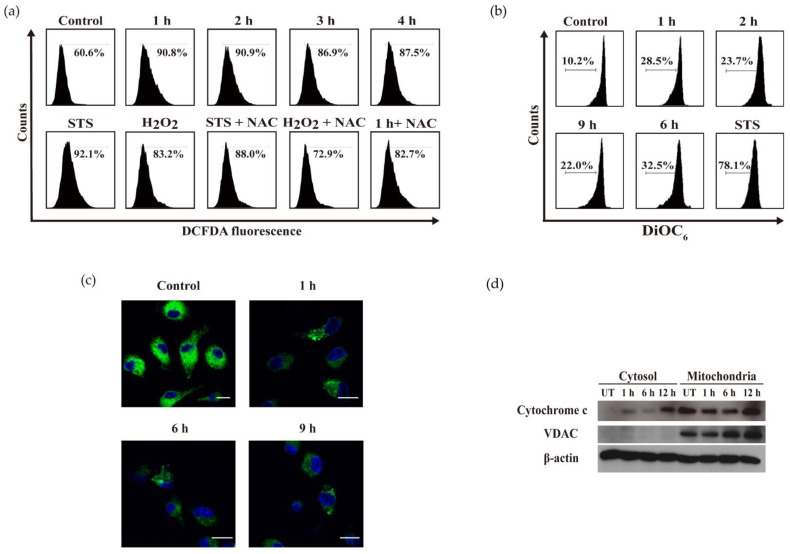
OH-dDHL induced ROS production and mitochondrial dysfunction in BMDMs. (**a**) BMDMs were treated with 50 µM OH-dDHL in a time-dependent manner; STS (500 nM) and H_2_O_2_ (850 µM) for 4 h were used as positive controls; NAC (10 mM) stained with CM-H_2_DCFDA for ROS analysis followed by flow cytometry. (**b**) BMDMs were treated with 50 µM OH dDHL and stained with 3,3′-dihexyloxacarbocyanine iodide DiOC_6_. Cells were analyzed using flow cytometry. (**c**) BMDMs were treated with 50 µM OH-dDHL and imaged with a confocal microscope using the FITC channel. Scale bar, 10 µm. (**d**) BMDMs were treated with 50 µM OH-dDHL in a time-dependent manner. Cytochrome c analyzed using Western blot in cytosol fraction and mitochondria fraction. UT; negative control.

The balance of intracellular ROS is tightly regulated by physiologic conditions; disrupting this balance can lead to excessive ROS production, oxidative damage including ER and mitochondria, and apoptosis [21]. We utilized CM-H_2_DCFDA and DiOC_6_, which are fluorogenic dyes, to determine the effects of OH-dDHL on cellular ROS production and mitochondrial membrane potential. As shown in Figure 5a, ROS generation by OH-dDHL resulted in a noticeable increase in fluorescence intensity after 1 h and the STS positive control and H_2_O_2_ increased it to a similar degree. The OH-dDHL-induced ROS generation was significantly reduced by the pre-treatment with NAC (ROS scavenger). The mitochondrial membrane potential was also analyzed and there was a slight decrease after the OH-dDHL treatment relative to the control, confirming the reduction in mitochondrial membrane potential using confocal microscopic analysis (Figure 5b,c). In addition, the release of cytochrome c from the mitochondria and their subsequent translocation to the cytosol were evaluated using the immunoblots of the mitochondrial- and cytosolic-fraction obtained from the OH-dDHL-treated BMDMs for 1 h, 6 h, and 12 h. It was observed that the cytochrome c levels in the cytosol were gradually increased up to 12 h after the OH-dDHL treatment (Figure 5d). These results support that OH-dDHL-induced cellular ROS and mitochondria dysfunction also activate apoptosis as an intrinsic signaling pathway associated with ER stress.

### 2.5. Effect of Lipid Raft and PON2 on Cell Viability of BMDMs

**Figure 6:**
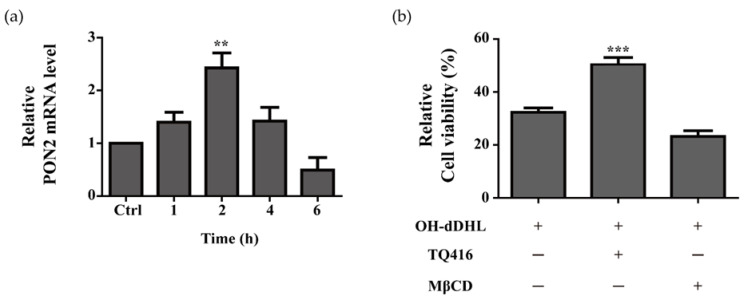
Effect of lipid raft and PON2 on BMDM cells treated with OH-dDHL. (**a**) BMDMs were treated with 50 µM OH-dDHL in a time-dependent manner. Relative expression of mRNA was measured using a real-time quantitative PCR. Gene expression was normalized with GAPDH. (**b**) Cell viability was determined using CCK assays. MβCD (2 µM) was pre-applied for 1 h and TQ416 (4 µM) was applied simultaneously. BMDMs were treated with 50 µM OH-dDHL for 3 h. Data are presented as mean ± SEM of three independent experiments. ***, *p* < 0.001, **, *p* < 0.01 vs. OH-dDHL.

Cholesterol in the plasma membrane, which acts as a receptor for AHL and paraoxonase-2 (PON 2), catalyzes the hydrolysis of AHL. Both of these mechanisms have been implicated in promoting AHL-induced cell death [28,29]. To investigate the functional significance of plasma-membrane cholesterol and the functional relevance of PON2 hydrolase to mediate the cellular damage caused by OH-dDHL in BMDM, the lipid raft inhibitor methyl-β-cyclodextrin (MβCD) and PON2 inhibitor ((1,2,4)triazolo (4,3-a)quinolone; (TQ416)) was used to deplete cholesterol and inhibit PON2 activity. The PON2 expression level in BMDMs with 50 µM OH-dDHL was markedly increased compared to those in the controls (Figure 6a). TQ416 inhibited the effects of cytotoxicity by OH-dDHL by >15% in BMDMs, whereas pre-treatment with MβCD did not affect the BMDMs. (Figure 6b). In the A549 cells, TQ416 significantly inhibited the cytotoxicity of OH-dDHL and MβCD inhibited it by >9% (Figure S3). Thus, the data demonstrate that the hydrolysis of PON2 induced OH-dDHL cytotoxicity and the fluidity of the cell membrane to lipophilic OH-dDHL permeability varies depending on the cell characteristics.

### 2.6. Pro-Inflammatory Cytokine Gene Expression Is up Regulated by OH-dDHL on BMDMs

**Figure 7:**
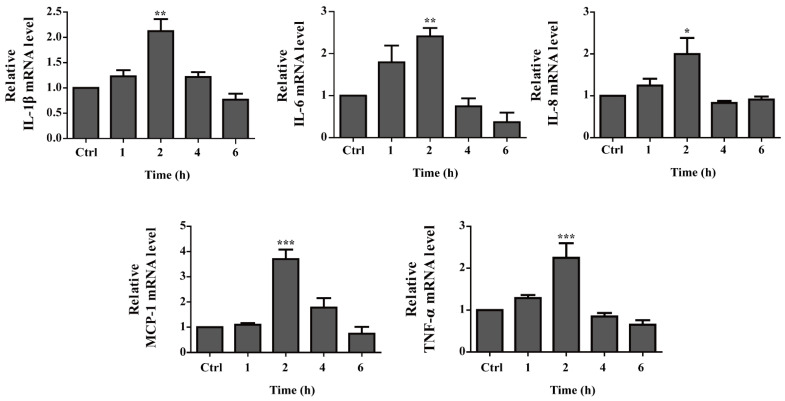
OH-dDHL-induced pro-inflammatory cytokine expression in BMDMs. BMDMs were treated with 50 µM OH-dDHL in a time-dependent manner. The relative expression of mRNA was measured using a real-time quantitative PCR. Gene expression was normalized with GAPDH. Data are presented as mean ± SEM of three independent experiments. ***, *p* < 0.001, **, *p* < 0.01. *, *p* < 0.05 vs. control.

Although several studies have suggested that AHL enhances the pro-inflammatory response, other studies have indicated that AHL provides anti-inflammatory effects, thus contributing to the establishment of a persistent infection [25,30]. To determine the effect of OH-dDHL on inflammatory signaling in BMDM, we confirmed the qPCR amplification efficiency of each primer and stable reference gene Cq values (Figure S4), and then measured the transcription of pro-inflammatory genes using a real-time quantitative PCR. The BMDMs were treated for 6 h in the presence or absence of OH-dDHL, the results of which are summarized in Figure 7. The TNF-α, IL-1β, IL-6, IL-8, and MCP-1 expression levels were markedly increased up to twofold compared to those in the control at an incubation time of 2 h, but then decreased to the level of the untreated control. These results indicate that OH-dDHL mediates the initial inflammatory response.

### 2.7. Lung Colonization and Virulence by A. nosocomialis

**Figure 8:**
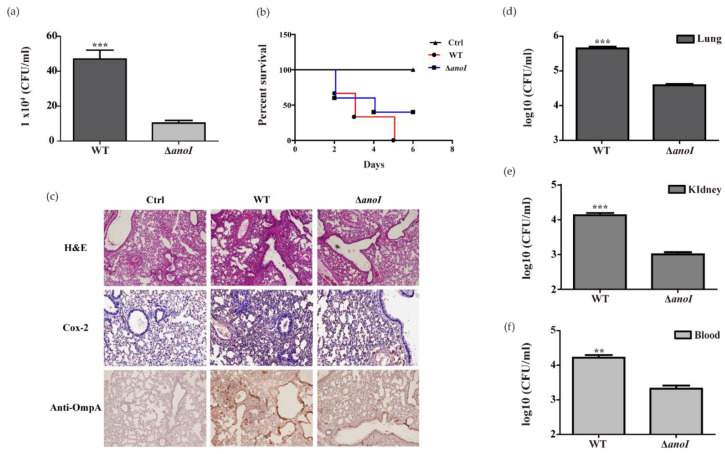
Lung colonization and virulence by *A. nosocomialis*. (**a**) BMDMs were infected with WT, Δ*anoI*, at an MOI of 100 for 6 h and incubated for 2 h in complete medium containing gentamicin, followed by additional incubation in complete medium for 16 h. Δ*anoI*. Data are presented as mean ± SEM of three independent experiments. (**b**) Survival curve of neutropenic BALB/c mice infected with *A. nosocomialis*. Neutropenic BALB/c mice were intratracheally infected with WT, Δ*anoI*, and their survival rate was monitored twice a day. (**c**) Histological analysis of lungs in the mice three days post-infection. Representative histopathological sections of hematoxylin and eosin-stained lungs from neutropenic BALB/c mice infected with *A. nosocomialis*. Anti-OmpA antibody labeled lung sections WT, Δ*anoI*. The lungs of mice infected with WT and Δ*anoI* showed areas of inflammatory cell infiltration. Magnification: ×100. (**d**–**f**) Mice were intratracheally infected with *A. nosocomialis* strains and the number of bacteria in the lungs, blood, and kidneys three days post-infection was determined. Statistical significance was checked using Student’s *t*-test. ***, *p* < 0.001, **, *p* < 0.01 compared with Δ*anoI.*

To further characterize the virulence of *A. nosocomialis*, we evaluated the intracellular bacteria number in infected BMDMs with wild-type and the *anoI*-deletion mutant. The intracellular bacteria number of the wild-type strain was significantly higher than that of the *anoI*-deletion mutant strain (Figure 8a). In addition, the intracellular *anoI*-deletion mutant strain was significantly decreased in A549 cells (Figure S5). In intratracheally infected BALB/c mice in the pneumonia model, the survival rate was observed for six days after infection. The wild-type strain showed a 50% survival rate after three days and 0% after five days. However, the mutant strain showed a 40% survival rate after six days (Figure 8b). The mice were sacrificed three days post-infection to observe their histopathological features. The *anoI*-deletion mutant strains showed significantly reduced histopathological features in which lymphocytes and macrophages had been recruited in the alveolar spaces. In addition, the pathological inflammation of the mice infected with the mutant strain was reduced compared to the wild-type strain.

We stained with anti-rabbit polyclonal OmpA for *A. nosocomialis* detection. The wild-type strains were found to have significantly more bacterial aggregates around the bronchus and the bronchoalveolar epithelium than the *anoI*-deletion mutant strains (Figure 8c). The *anoI*-deletion mutant strain showed decreased CFUs compared to the wild-type strain in the lung, and the dissemination from the lungs to the kidney had also declined (Figure 8d–f). Our results show that OH-dDHL production plays an important role in pathogenicity, such as the colonization of *A. nosocomialis* in the early stages of cell invasion and infection.

## 3. Discussion

Pathogenic bacteria have important interactions with immune cells at the site of infection and have evolved to survive and replicate by subverting immune responses [31]. The innate immune response mainly consists of the protective abilities of neutrophils and macrophages [15]. In an *Acinetobacter* infection, monocytes and macrophages are activated in an early stage of infection; in particular, alveolar macrophages in early infections react first at the site of infection before recruiting neutrophils [32,33]. While the innate immune response plays an important role in inducing apoptosis to eliminate the bacteria, the bacteria control apoptosis using various factors, including toxins and virulence factors, for invasion into the bloodstream or dissemination into other organs [34,35]. For example, Staphylococcal pore-forming toxins (PFTs), *E. coli* outer membrane vesicles (OMVs), *Shigella* toxin, *P. aeruginosa* toxin, and QS molecules induce macrophage death [36,37]. In this study, we have demonstrated that the OH-dDHL of *A. nosocomialis* induces ER- and mitochondria-mediated apoptosis of BMDMs.

Short acyl chain QS molecules, including *N*-butanoyl-l-homoserine lactone (BHL), *N*-(3-oxo-hexanoyl)-L-homoserine lactone (OHHL), *N*-hexanoyl-DL-homoserine lactone (C6-HSL: HHL), and *N*-decanoyl-DL-homoserine lactone (C10-HSL: DHL) showed no cytotoxicity effect on the host cells [24]. We confirmed that DHL, HHL, and OH-dDHL produced by *Acinetobacter* spp. are cytotoxic. Exclusively, OH-dDHL decreased the cell viability of THP-1, HEp-2, A549, and BMDMs (Figure 1). At a concentration of 50 µM or more, cell viability decreased in a time-dependent fashion (Figure 2 and Figure S1). In addition, we demonstrated that OH-dDHL induced apoptosis through a caspase-dependent pathway (Figure 3). These results are consistent with a previous report that 3-oxo-C12-HSL induces the apoptosis of immune cells and human colon cancer cell lines [30,38].

In ER stress, the ER stress markers IRE1, ATF6, and PERK are activated by unfolded protein accumulation, which activates molecules such as downstream p-eIF2α and CHOP, which are major markers of the ER stress-mediated mitochondrial pathway of apoptosis involving oxidative stress and an impaired mitochondrial function [22]. The Ca^2+^ released from the ER triggered by an ER stressor is directly related to the mitochondria, activates caspase-12, and induces the ER stress-mediated mitochondrial pathway of apoptosis through oxidative stress and activated Bax and Bak [23]. However, caspase-12 directly activates caspase-9 and -3, independent of the mitochondrial pathway [23,39]. Although we could not detect the activation of phosphate PERK, we demonstrated the activation of p-eIF2α by OH-dDHL. We observed ER stress with transmembrane protein (IRE1 and ATF6a p50) aggregation induced by OH-dDHL in BMDMs and subsequent CHOP and caspase-12 activity. Ca^2+^ was released into the cytosol immediately in treated BMDMs (Figure 4). CHOP and caspase-12 were activated within 15 min in BMDMs by OH-dDHL (data not shown). Intrinsic pathway apoptosis is involved in mitochondria dysfunction such as increased outer mitochondrial membrane permeabilization [21,22]. The collapse of the mitochondrial membrane potential (ΔΨm) and the consequent activation of Bax and Bak by an ROS such as H_2_O_2_ results in cytochrome c release, leading to caspase-9 and -3 activation [21]. It has been previously reported that 3-oxo-C12-HSL promotes the release of Ca^2+^ from the ER in nonpolarized airway epithelial cells and triggers apoptosis by the Bak/Bax-independent release of cytochrome c and both the extrinsic and intrinsic pathways [27,40,41]. Although the precise molecular mechanism of OH-dDHL action and the activation of Bax and Bak on mitochondria is undetermined, our study showed that OH-dDHL induces an apoptotic signaling pathway that activates caspase-9, and -3 by directly acting on mitochondria to release cytochrome c and ROS generation. Since the effect of ER stress on ΔΨm occurs within an hour after ER Ca^2+^ depletion, it is OH-dDHL-induced ER Ca^2+^ release that will indirectly cause the depolarization of ΔΨm. Therefore, it appears that ER stress signaling is indirectly involved in the depolarization of OH-dDHL-mediated ΔΨm. Thus, these data indicate that OH-dDHL triggers apoptosis through both the ER- and mitochondria-mediated pathways.

The lipophilic *N*-acyl-homoserine lactone can easily infiltrate into host cells [30]. Lipid rafts are involved in cell signaling, LPS signaling in macrophages, and pathogen phagocytosis [30,42]. It was previously reported that the epithelial barrier dysfunction by 3-oxo-C12-HSL is associated with lipid rafts and the lipid rafts inhibitor, MβCD, inhibited the loss of the TJ protein and the permeability of the Caco-2 monomer by 3-oxo-C12-HSL [29]. However, MβCD has a minor effect on LS174T cells [30]. PON2 with lactonase activity has reportedly induced ER stress and Bak/Bax-independent apoptosis by a hydrolyzed 3-oxo-C12-HSL-acid accumulation in cells [43]. TQ416 inhibits PON2 activity and the cytotoxicity of 3-oxo-C12-HSL on LS174T cells [30]. As shown in Figure 6 and Figure S3, TQ416 inhibits the cytotoxicity of OH-dDHL on BMDMs and A549 cells. MβCD inhibited the cytotoxicity of OH-dDHL on A549 cells and had no effect on BMDMs. Considering the increased cell viability when PON2 activity was inhibited by TQ416, it is thought that OH-dDHL directly induces caspase-8 activity, mitochondrial dysfunctions, and ROS generation. This denotes that studies are needed on the upstream stress response other than OH-dDHL acidification. Our results indicated that OH-dDHL cytotoxicity by lipid raft suppression occurs due to differences in the characteristics of epithelial cells and phagocytic cells, and OH-dDHL acid accumulation by PON2 induces a cellular response consistent with hydrolyzed 3-oxo-C12-HSL acid accumulation.

In our study, we have demonstrated that OH-dDHL modulates the pro-inflammation cytokines MCP-1, IL-8, TNF-α, IL-1β, and IL-6 (Figure 7) and we have shown that caspase-8 is activated by OH-dDHL (Figure 3). The extrinsic apoptosis pathway is mediated through caspase-8 by TNF and TRAIL [21]. The OMVs of *A. baumannii* and *A. nosocomialis* trigger pro-inflammatory cytokines, including IL-1β, IL-6, MIP-1a, and MCP-1 [44]. Macrophage cells infected with *A. baumannii* reportedly secrete the inflammatory cytokines MIP-2, IL-6, and TNF-α [32]. Furthermore, infected mice have increased pro-inflammation cytokines IL-1β, IL-6, MIP-2, and TNF-α [33]. The application of 3-oxo-C12-HSL to LS174T cells reportedly induces the pro-inflammation cytokines IL-1β and IL-8 [30], which elicit TNF-α in resting RAW264.7 [25]. In contrast, 3-oxo-C12-HSL suppresses the pro-inflammation cytokine TNF-α and increases the anti-inflammation cytokine IL-10 in stimulated RAW 264.7 [25]. We suggest that the inflammatory cytokines released by OH-dDHL are involved in the early stages of *Acinetobacter* infection, facilitating interference with the host response and dispersal to other sites of replication.

As noted above, the QS system is involved in biofilm formation; in particular, biofilms are important in colonization and adherence for host cell infection in the early stages [45]. Mutations in the autoinducer synthase LuxI reduce biofilms, which has been demonstrated in *H. alvei*, *A. baumannii*, and *P. fluorescens* [6,46,47]. In well-studied *P. aeruginosa*, it has been reported that *lasI* mutant strains in vivo showed less bacteria pathogenicity than wild-type strains [48]. We demonstrated that the *anoI*-deletion mutant had a decreased invasion and adhesion (Figure 8 and Figure S5). The *anoI*-deletion mutant showed significantly less virulence and dissemination in mouse models and immune responses in the lungs were also reduced (Figure 8). Our results suggest that the activity of OH-dDHL may be an important factor for invasion and proliferation in the early stages of infection. Although the pathogenicity regulation of the *A. nosocomialis* QS system was confirmed in this study, the QS concentration of *Acinetobacter* spp. in vivo has not been demonstrated. A previous study in cystic fibrosis patient sputum reported QS molecules of *P. aeruginosa* 1–20 nM [25] were found at low concentrations in vivo. However, QS molecules of *P. aeruginosa* are observed at 600 µM in biofilms [25,49]. Previous studies have suggested that exposure to high concentrations of QS molecules is required for an appropriate biofilm formation in the host [49]. Thus, we speculate that *Acinetobacter* spp. will enable similar events. However, the exact QS concentration of *Acinetobacter* spp. in biofilms and in vivo should be clearly investigated.

In summary, we demonstrated that OH-dDHL induced ER- and mitochondria-mediated apoptosis and the *anoI*-deletion mutant had decreased virulence in vivo. Overall, our data may provide new insights into the mechanism of host cell apoptosis by the QS molecule OH-dDHL and contribute to our understanding of the interactions between *A. nosocomialis* and host cells in the early stages of infection.

## 4. Materials and Methods

### 4.1. The Bacterial Strains, Culture Conditions

The *A. nosocomialis* ATCC 17903 type strain and *anoI*-deletion mutant strains were grown separately in Luria-Bertani (LB) broth containing 1.5% (wt/vol) agar at 37 °C. The *anoI*-deletion mutant strain was constructed using the conjugation method [9,50,51]. All bacterial cells were delivered without antibiotics at a density of 1.0 x 10^8^ cfu mL^−1^ for the infection

### 4.2. Reagent and Antibody

Synthetic *N*-hexanoyl-DL-homoserine lactone (C6-HSL), *N*-decanoyl-DL-homoserine lactone (C10-HSL), *N*-(3-hydroxy dodecanoyl)-DL-homoserine lactone (OH-dDHL), tunicamycin, thapsigargin, hydrogen peroxide 30% (wt/vol) (H_2_O_2_), *N*-Acetyl-l-cysteine (NAC), and staurosporine were purchased from Sigma-Aldrich (St. Louis, MO, USA). Antibodies with anti-caspase-3, anti-caspase-8, anti-cytochrome c, anti-PARP, anti-β-actin, anti-caspase-9, anti-VDAC, anti-CHOP, anti- caspase-12, anti-p-eIF2α, anti-IRE1α, and anti-BIP were purchased from Cell Signaling Technology (Danvers, MA, USA). Anti-p-IRE1α was purchased from Thermo Fisher Scientific (Waltham, MA, USA). Anti-ATF 6α, anti-COX-2, and methyl-β-cyclodextrin (MβCD) were purchased from Santa Cruz Biotechnology (Paso Robles, CA, USA). Horseradish peroxidase-conjugated goat anti-rabbit IgG and goat anti-mouse IgG were purchased from Abcam (Cambridge, MA, USA). The pan-caspase inhibitor (Z-VAD-FMK), caspase-3 inhibitor (Z-DEVE-FMK), and caspase-9 inhibitor (Z-LEHD-FMK) were purchased from R&D Systems (Minneapolis, MN, USA). The paraoxonase 2 (PON2) inhibitor ((1,2,4)trizolo(4,3–a)quinolones; TQ416) was purchased from Maybridge (Cornwall, UK).

### 4.3. Cell Culture

A549 cells (human alveolar epithelial cell line; ATCC CCL-185) and HEp-2 cells (human laryngeal epithelial cell; ATCC CCL23) were cultured in Dulbecco’s Modified Eagle Medium (DMEM; Welgene, Daegu, Korea) supplemented with 10% fetal bovine serum (FBS; Welgene, Daegu, Korea) and 1% antibiotic–antimycotic solution (Welgene, Daegu, Korea) at 37 °C in 5% CO_2_. THP-1 cells from human monocytic cells were grown in RPMI 1640 medium (Welgene, Daegu, Korea) and supplemented with 10% FBS, 1% nonessential amino acid, and 1 mM sodium pyruvate (Sigma-Aldrich, St. Louis, MO, USA), and 1% antibiotic–antimycotic solution at 37°C in 5% CO_2_. Bone marrow-derived macrophages (BMDMs) were generated by flushing bone marrow cells from the femurs and tibias of 6–8-week-old female C57BL/6 mice, cultured for four days in DMEM, and supplemented with 10% FBS, 1% antibiotic–antimycotic solution, and 25 ng/mL mouse macrophage colony-stimulating factor (M-CSF) (R&D Systems, Minneapolis, MN, USA) at 37 °C in 5% CO_2_.

### 4.4. Determination of Cell Viability

The BMDMs’ viability was determined using the Cell Counting Kit-8 (CCK-8; Dojindo Laboratories, Gaithersburg, MD, USA). Cells were sub-cultured into 96-well microplates at 80% confluency at 37 °C. The cells were treated with OH-dDHL in a time- and dose-dependent manner and pretreated with MβCD inhibitors prior to OH-dDHL treatment, while TQ416 inhibitors were applied simultaneously. The viability of the cells was measured at 450 nm 1 h after treatment with CCK-8. The data are expressed as the percentage of viable cells relative to the untreated control cells, which was calculated using the absorbance ratio.

### 4.5. Apoptosis Analysis

Apoptotic cells were assessed via staining with an Annexin V/Propidium iodide (PI) staining kit in accordance with the manufacturer’s instructions (BD Biosciences, San Diego, CA, USA). In brief, the cells were harvested and incubated with FITC-conjugated Annexin V and PI in the dark for 15 min. Analysis of the stained cells was performed on an FACS Canto II (Becton-Dickinson, San Jose, CA, USA) with FACS Diva and the results were analyzed using FlowJo software (Tree Star, Ashland, OR, USA).

### 4.6. Immunofluorescence Microscopic Analysis

BMDMs were seeded (1 × 10^6^ cells/mL) onto glass coverslips in 12-well plates and treated with OH-dDHL for the indicated times. Nuclear changes in the cells were analyzed by staining with 4ʹ, 6-diaminido-2-phenylindole (DAPI). The BMDM cells were fixed with 4% paraformaldehyde and incubated with a Vectashield mounting medium containing DAPI (Vector Laboratories, Burlingame, CA, USA) overnight in the dark at 4 °C. The stained cells were observed using an Olympus BX50 fluorescence microscope (Olympus Optical Co., Hamburg, Germany).

### 4.7. Measurement of DNA Fragmentation by Cell Death Detection ELISAplus Kit

The measurement of DNA fragmentation was performed in accordance with the manufacturer’s instructions for the Cell Death Detection ELISA^PLUS^ kit (Roche Applied Science, Mannheim, Germany). First, BMDM cells were seeded in 96-well flat-bottom culture plates. After the cells were treated with OH-dDHL in a time-dependent manner, the supernatant was transferred to a 96-well microtiter plate, and the absorbance was measured using an ELISA plate reader (SpectroMAX, CA, USA) at 405 nm. The ratio of DNA fragmentation was expressed as the enrichment of DNA fragments in the treated sample:the DNA fragments in the control.

### 4.8. Western Blot Analysis

Cells were washed with cold PBS and lysed with RIPA lysis buffer (150 mM NaCl, 1% Triton X-100, 0.1% SDS, 1% sodium deoxycholate, 50 mM Tris-HCl pH 7.5, 2 mM EDTA) containing protease inhibitor cocktail (Halt^TM^, Pierce, Rockford, IL, USA) and 1 mM phenylmethylsulfonyl fluoride (PMSF) for 15 min on ice. The cell lysates were cleared by centrifugation and quantified using a Bradford assay (Bio-Rad, Hercules, CA, USA). An equal amount of each sample was separated by 8% or 15% SDS-PAGE, followed by an electrotransfer onto a PVDF membrane (EMD Millipore, Danvers, MA, USA). The blots were blocked in 0.05% Tween 20 containing 5% skim milk and incubated with primary antibodies. The proteins were visualized by incubation with appropriate horseradish peroxidase-conjugated secondary antibodies, followed by enhanced chemilluminescence (WesternBright ECL kit; Advanstar, San Jose, CA, USA) according to the manufacturer’s instructions.

### 4.9. Measurement of ROS Production

The intracellular hydrogen peroxide levels were measured via staining with the oxidant-sensitive fluorescent probe CM-H_2_DCFDA (5-(and-6)-chloromethyl-2′,7′-dichlorodihydrofluorescein diacetate, acetyl ester) (Molecular Probes, Eugene, OR, USA). The cells were incubated with OH-dDHL and then stained with CM-H_2_DCFDA for 30 min at 37 °C in the dark. The stained cells were immediately analyzed using the FACS Canto II with FACS Diva, and the results were analyzed using FlowJo software.

### 4.10. Measurement of Mitochondria Membrane Potential (ΔΨm) and Confocal Microscopic Analysis

BMDMs were seeded onto glass coverslips in 12-well plates and treated with OH-dDHL. Afterward, the cells were fixed with 4% paraformaldehyde (PFA; Sigma-Aldrich, St. Louis, MO, USA) and incubated with 3,3′-dihexyloxacarbocyanine iodide (DiOC_6_(3); Molecular Probes) at 37°C for 15 min in the dark. The stained cells were visualized using a Leica DMi8 confocal microscope (Leica Microsystems Ltd., Wetzlar, Germany). The mitochondrial membrane potential was determined by flow cytometer analysis using DiOC_6_(3) staining. The harvested cells were incubated for 15 min at 37 °C and then analyzed with a NovoCyte Flow Cytometer (ACEA Biosciences, Inc., San Diego, CA, USA). The percentages of the cells with membrane depolarization were calculated using FlowJo software (Tree Star, Ashland, OR, USA).

### 4.11. Mitochondria Fractionation

BMDMs were treated with OH-dDHL. The adherent and detached cells were harvested, and the mitochondrial fraction was carried out by using a Mitochondrial Isolation Kit (Pierce, Rockford, IL, USA), according to the manufacturer’s instructions.

### 4.12. Measurement of Intracellular Calcium (Ca^2+^)

A cell-permeable Fluo-8 AM (Abcam, Ltd., Cambridge, UK) was used to evaluate the effect of OH-dDHL on the intracellular calcium mobilization in the BMDMs. The experiments were performed according to manufacturer’s instructions with a minor modification. Briefly, BMDMs were cultured overnight with growth medium in 96-well plates. Then, cells were washed with Hanks’ balanced salt solution to minimize background fluorescence and interference with serum. Cells were incubated with Fluo-8 AM for 1 h in HBSS prior to treatment with OH-dDHL for the indicated dose. The cytoplasmic calcium level was documented by the fluorescence intensity under an SpectroMAX ELISA plate reader (Molecular Devices, San Jose, CA, USA). For the confocal microscopic analysis, cells were seeded onto glass coverslip in 12-well plates and treated with OH-dDHL in a dose-dependent manner. Cells were incubated with Fluo-8 AM for 1 h at 37 °C in the dark and further fixed with 4% PFA. The coverslips were washed and mounted on microscope slides by using Vectashield mounting medium containing DAPI and stored at 4 °C in the dark. The stained samples were viewed with a Leica DMi8 confocal microscope (Leica Microsystems Ltd., Wetzlar, Germany).

### 4.13. RNA Extraction, Revers Transcription, and Real-Time Quantitative PCR (RT-qPCR)

Total RNA was extracted from OH-dDHL treated in BMDM using the RNeasy Mini kit (Qiagen, Venlo, Netherlands) in accordance with the manufacturer’s protocol. cDNA was amplified by random hexamers using a Reverse Transcription premix (ElpisBio, Daejeon, Korea). Table 1 lists the primers used in the Polymerase Chain Reactions (PCRs). Quantitative PCR was carried out with cDNA in the CFX96 Real-time PCR detection system (Bio-Rad Hercules, CA, USA) with Prime Q-Mastermix with SYBR Green (GeNet Bio, Choenan, Korea) according to following conditions: (a) initial denaturation: 95 °C for 10 min, (b) 40 cycles including denaturation 30 sec, annealing: 58 °C for 30 sec, extension: 72 °C for 30 sec. The expression levels of the genes were normalized to GAPDH using the comparative quantitation cycle (Cq) method (2^−∆∆Cq^), as described by the CFX96 Real-time PCR detection system (CFX Maestro™ Software, Bio-Rad Hercules, CA, USA). All RT-qPCR reactions were run in triplicate, and a non-template control was used for each run.

### 4.14. Bacterial Invasion Assay

BMDM cells were seeded (1 × 10^6^ cells/mL) in 12-well plates containing DMEM supplemented with 10% FBS and incubated at 37 °C in 5% CO_2_ for 24 h. Cultures of *A. nosocomialis* WT and *anoI*-deletion mutant strains were grown in LB broth overnight at 37 °C. Cells were infected with the bacterial cultures for 6 h at an MOI of 100. After incubation at the indicated condition, the cells were washed three times with DPBS before adding DMEM-FBS (10%) containing 300 μg/mL of gentamicin to kill all external bacteria. Fresh DMEM medium was added and incubated for another 16 h. The cells were washed with DPBS and lysed with 0.25% Triton X-100 at 37 °C for 20 min. Dilutions from each well were plated on LB agar plates and the colonies were counted to quantify the bacteria that had survived intracellularly.

### 4.15. Animal Experiments

Mice were purchased from Nara Biotech, Korea, and the animals were maintained under specific-pathogen-free conditions in accordance with the guidelines of the Institutional Animal Care and Use Committee (IACUC) of Chungnam National University (permission number: CNU-01182, Approval date: 26, Dec. 2018). The animal experiments were carried out in accordance with the Korean Food and Drug Administration guidelines. Seven-week-old female BALB/c mice were induced to neutropenic mice via intraperitoneal injections of cyclophosphamide (150 mg/kg) on days 4 and 1 prior to the injection of bacterial cells. The mice were anesthetized with tribromoethanol and groups of five were injected intratracheally with 50 µL of 1 × 10^8^ CFU/mL of bacteria. The control mice groups were injected with 50 µL of sterilized PBS (pH 7.4). The mice were sacrificed three days after bacterial challenge and their lungs were excised to check for colonization efficiency. The lungs were washed with sterilized PBS and then homogenized. The cell suspension was serially diluted and spotted onto LB agar plates to count the bacteria. For histological analysis, the tissues were stained with hematoxylin/eosin, anti-COX-2, or immunohistochemical staining with OmpA antibody to visualize the bacteria.

### 4.16. Data Analysis and Statistics

Averages and standard errors of the means (SEM) were calculated from at least three independent experiments. All data were analyzed using unpaired Student’s *t*-tests, one-way ANOVAs, and Tukey’s multiple comparison test using statistical software (Graph Pad Prism Software, version 5.01; GraphPad Software, San Diego, CA, USA). Differences between experimental groups were considered to be significant at a *p*-value of < 0.05, ** *p* < 0.01, and *** *p* < 0.001.

## Figures and Tables

**Table 1:** Gene names and primer sequences (mouse) used in this study.

Gene Name	Forward Primer	Reverse Primer
TNF-α	AGGCACTCCCCCAAAAGATG	GTAGACAGAAGAGCGTGGTGG
IL-1β	CAACAAGAGCTTCAGGCAGG	TGCTCATGTCCTCATCCTGG
IL-6	GTTGCCTTCTTGGGACTGAT	GGTATAGACAGGTCTGTTGG
IL-8	GCTACGATGTCTGTGTATTC	TCACTTCCTTTCTGTTGCAG
MCP-1	CCACTCACCTGCTGCTACTC	ACAGCTTCTTTGGGACACCT
PON2	CTAATGGACAGAGGCTCTTC	TACACCGTTGTCACTGATGG
GAPDH	GTTCCAGTATGACTCCACTC	GTCTCGCTCCTGGAAGATGG

## Data Availability

All data supporting the findings of this study are available within the paper and its supplementary materials published online.

## Supplementary Materials

The following are available online at https://www.mdpi.com/article/10.3390/ijms22147565/s1.
