# Supplementary material for: N-3-Hydroxy Dodecanoyl-DL-homoserine Lactone (OH-dDHL) Triggers Apoptosis of Bone Marrow-Derived Macrophages through the ER- and Mitochondria-Mediated Pathways
Source: Int J Mol Sci. 2021 Jul 15;22(14):7565. doi: 10.3390/ijms22147565 (PMC8305837; doi:10.3390/ijms22147565)
Supplement: Supplementary file 1 [file ijms-22-07565-s001.zip › ijms-1283306-supplementary.pdf]

International Journal of
Molecular Sciences
MDPI

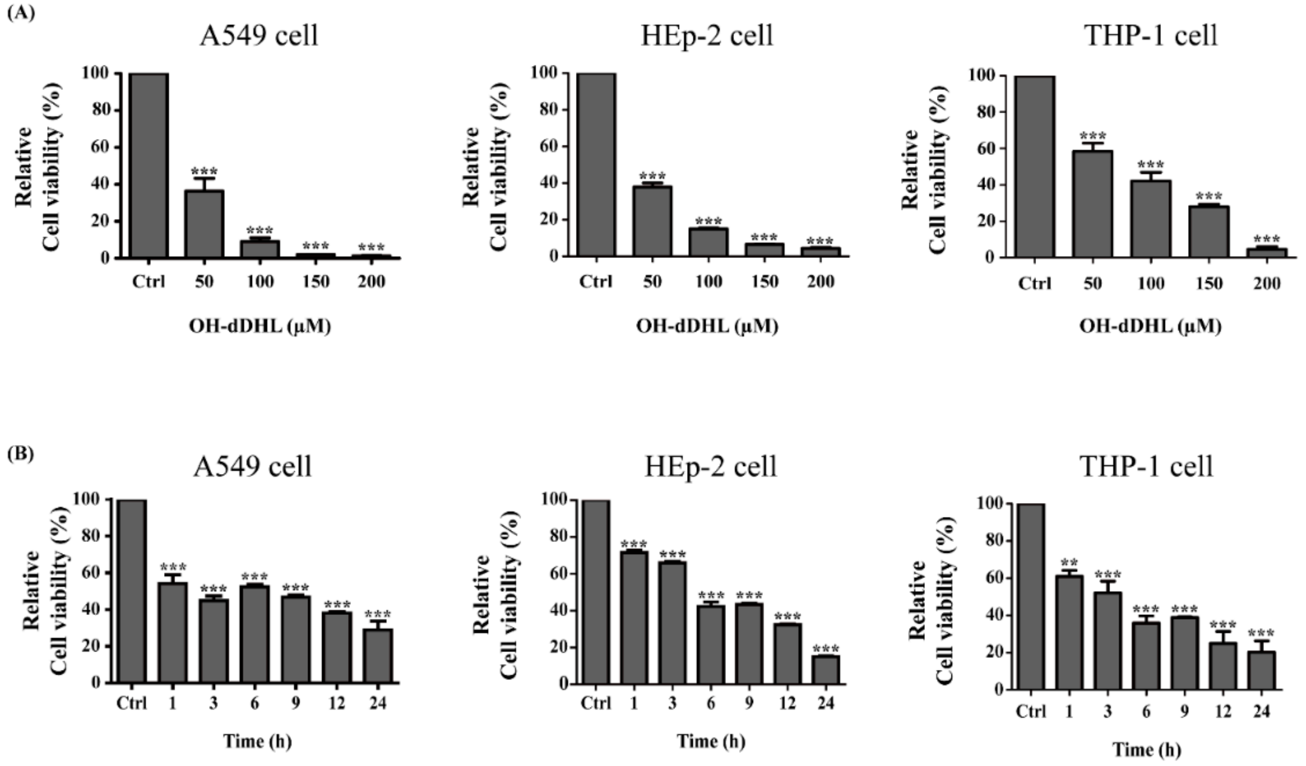

Figure S1. OH-dDHL decreases the cell viability of cultured cells. (A) A549, HEp-2, and THP-1 cells were treated with OH-dDHL dose-dependently for 24 h and cell viability was measured by CCK assays. (B) A549, HEp-2, and THP-1 cells were treated with 100 μM OH-dDHL in a time-dependent manner. Data are presented as means ± SEM of three independent experiments. ***, $p < 0.001$, **, $p < 0.01$ vs. control.

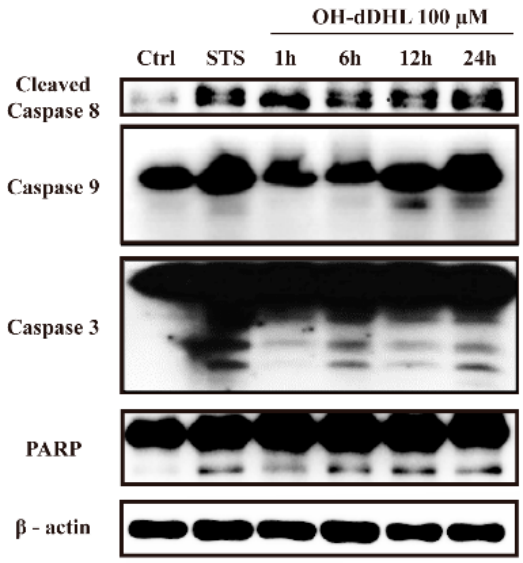

Figure S2. OH-dDHL activates of caspase in A549 cells BMDMs were treated in a time-dependent manner. Staurosporine (STS) was used as a positive control. Caspase-8, -9, and -3, and PARP were analyzed by western blot. Ctrl; negative control.

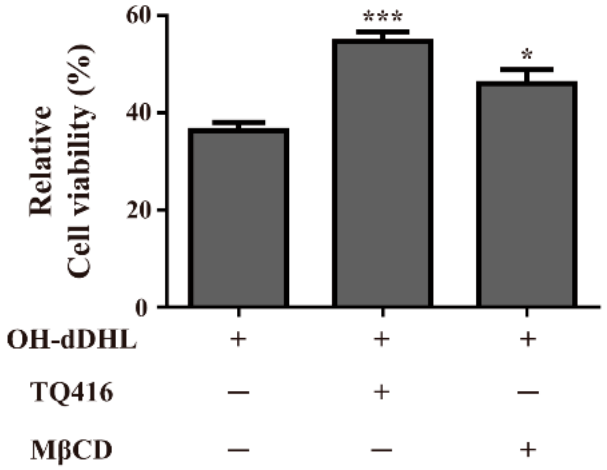

Figure S3. Effect of lipid raft and PON2 on the cell viability of A549 cells. Cell viability was determined by CCK assays. MβCD (4 μM) was preapplied for 1 h and TQ416 (4 μM) was simultaneously applied. BMDMs were treated with 100 μM OH-dDHL for 3 h. **, $p < 0.01$. *, $p < 0.05$ vs. control.

(a)

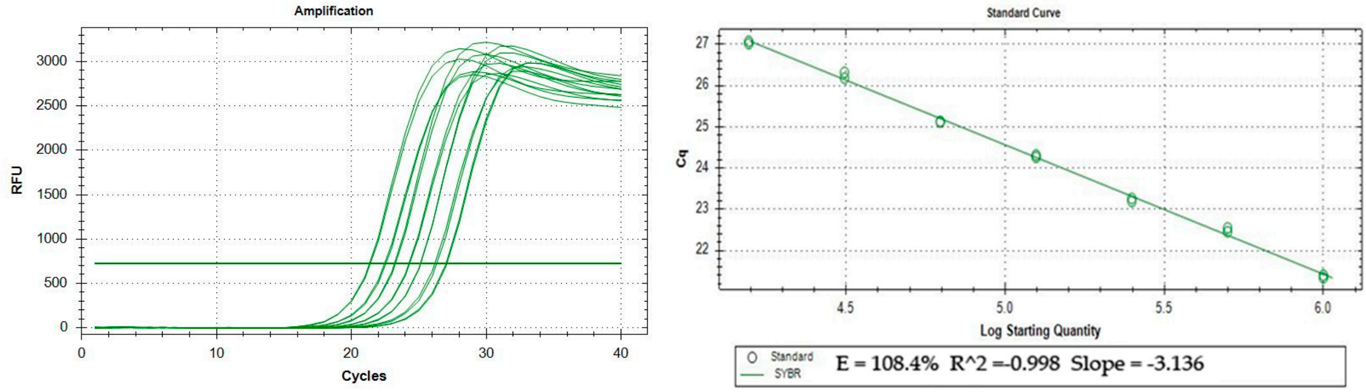

(b)

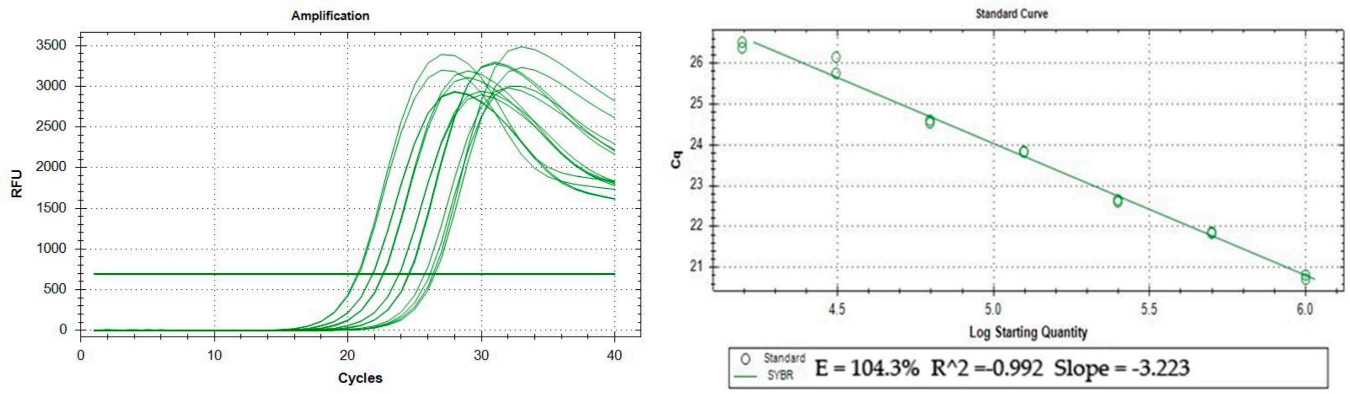

(c)

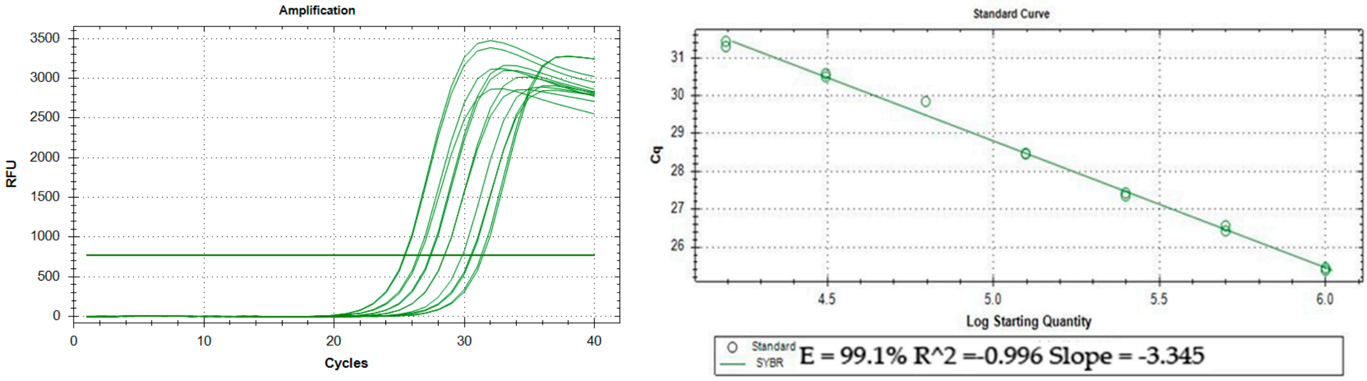
Amplification
RFU
Cycles
Standard Curve
Cq
Log Starting Quantity
Standard
SYBR
E = 99.1% R^2 =-0.996 Slope = -3.345

(d)

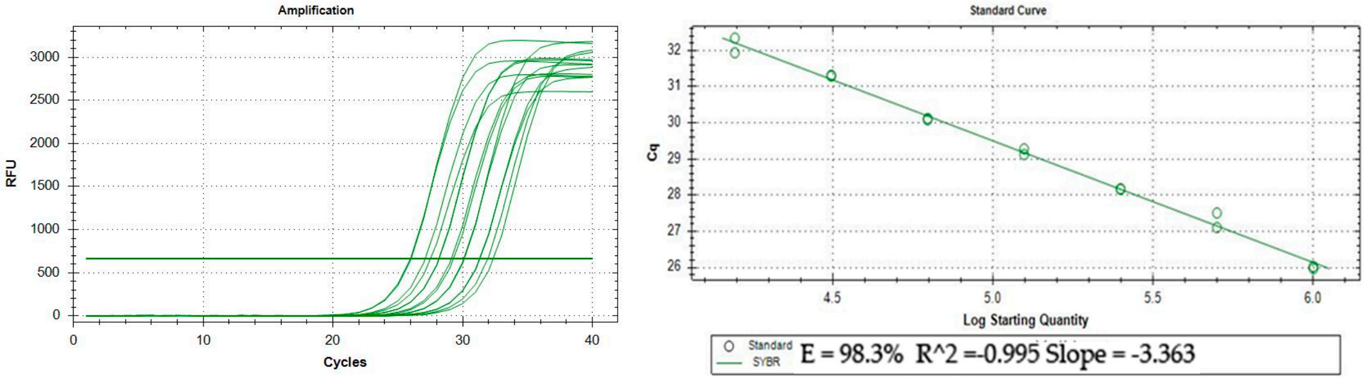
Amplification
RFU
Cycles
Standard Curve
Cq
Log Starting Quantity
Standard
SYBR
E = 98.3% R^2 =-0.995 Slope = -3.363

(e)

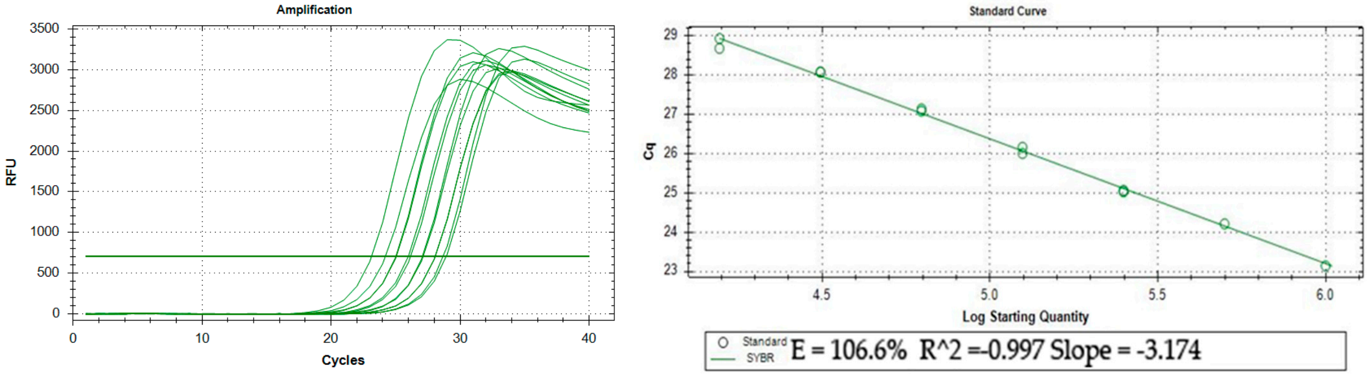
Amplification
RFU
Cycles
Standard Curve
Cq
Log Starting Quantity
Standard
SYBR
E = 106.6% R^2 =-0.997 Slope = -3.174

(f)

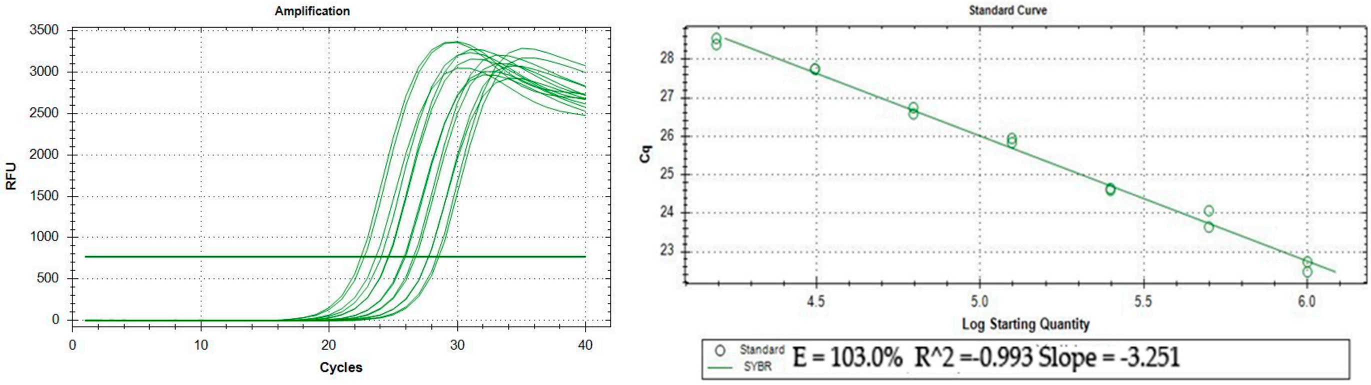

(g)

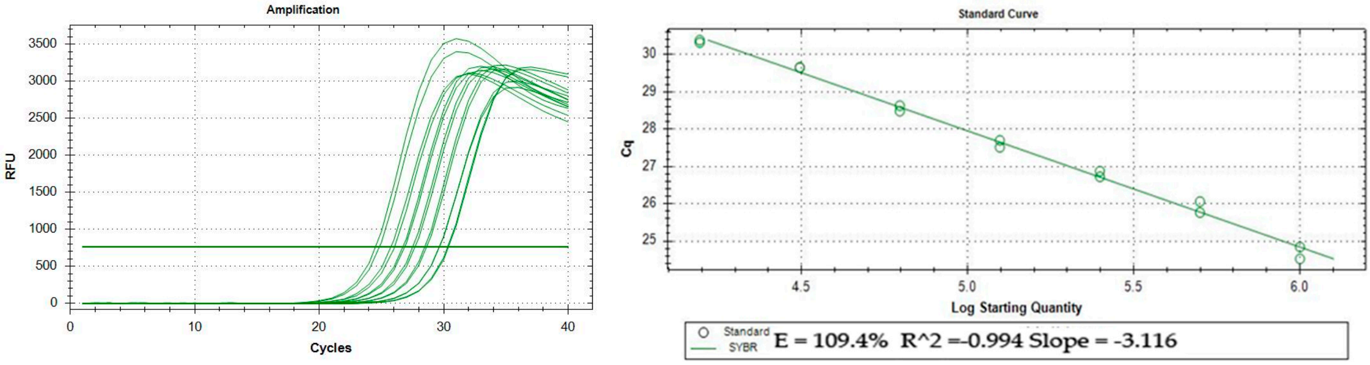

(h)

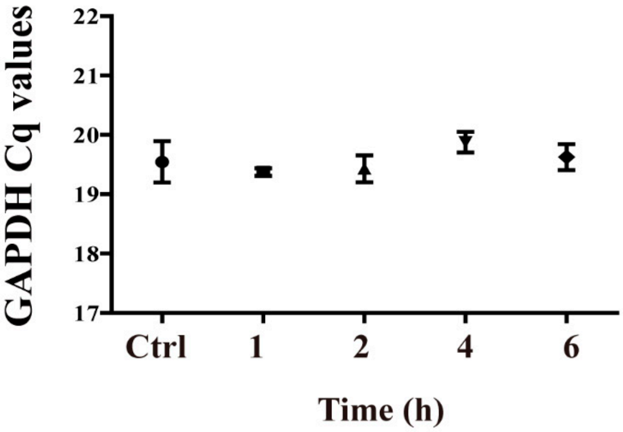

Figure S4. The amplification and standard curve of cDNA-based RT-qPCR. The graphs illustrate the efficiency and sensitivity for amplification of genes of interest and reference genes from 2-fold serially diluted cDNA. (a) GAPDH, (b) IL-1β, (c) IL-6, (d) IL-8, (e) MCP-1, (f) TNF-α, (g) PON2. (h) Plot comparison of mean Cq values by 50 μM OH-dDHL.

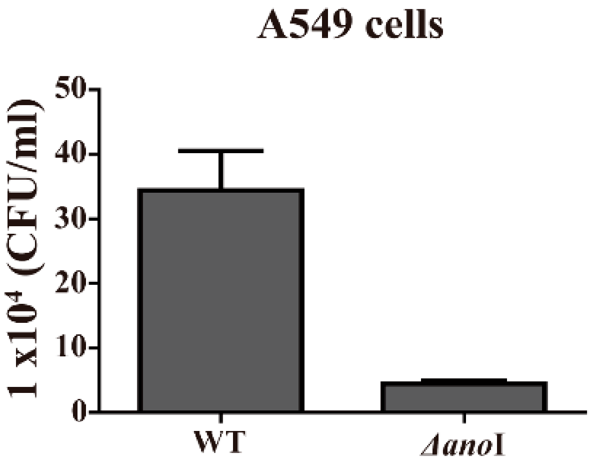

Figure S5. Quorum sensing system of *ΔanoI* reduces the ability of invasion in A549 cells. BMDMs were infected with WT and *ΔanoI* at an MOI of 100 for 6 h and incubated for 2 h in complete medium containing gentamicin, followed by additional incubation in complete medium for 16 h. *ΔanoI*. Data are presented as means ± SEM of three independent experiments.
